# Understanding the factors that shape patient choices in bringing a claim for clinical negligence against the NHS in England: a scoping review

**DOI:** 10.3389/frhs.2025.1696964

**Published:** 2026-01-08

**Authors:** Naomi Assame, Susan Greenhalgh, John Tingle, Gillian Yeowell

**Affiliations:** 1 Department of Health Professions, Manchester Metropolitan University, Manchester, United Kingdom; 2 Birmingham Law School, University of Birmingham, Birmingham, United Kingdom

**Keywords:** claim, clinical negligence, England, NHS, patient choice

## Abstract

**Background:**

Improving patient experience is a prominent theme of the National Health Service (NHS) 10-Year Health Plan for England including the need to improve patient experience of clinical negligence claims. Understanding the factors that shape patient choices in bringing a claim for clinical negligence is an important aspect of patient experience that has the potential to provide crucial insight that could inform the future reform of the clinical negligence process in England. This scoping review aimed to identify the key concepts within the limited research exploring the factors that shape patient choices in bringing a claim for clinical negligence against the NHS in England and identify where gaps in the research may exist.

**Methods:**

To address this knowledge gap, a methodological framework for conducting scoping reviews was applied. Search strategies were developed using selected keywords and index terms. Relevant published literature was identified by applying the search strategy to the peer-reviewed databases MEDLINE, Cumulative Index to Nursing and Allied Health Literature (CINAHL) and Westlaw. Reference lists of relevant publications were searched to identify relevant research and academic policy. All studies identified were charted, and the results were presented as a narrative synthesis.

**Results:**

Two main themes were identified from the 17 included records. These themes were ‘experience of harm’ and ‘accessibility of compensation for clinical negligence’.

**Conclusion:**

How an NHS organisation responds to harm can shape patient choices in bringing a claim for clinical negligence. However, this scoping review identified the limited consideration given to how current law and policy, organisational cultures, social determinants of health and health inequalities may shape patient choices in bringing a claim for clinical negligence. Furthermore, this scoping review has identified that empirical research has given no consideration to the role of social media or Artificial Intelligence (AI) in shaping patient choices in bringing a claim for clinical negligence. Research considering these factors is vital to improve patient experience of the clinical negligence process in England and has the potential to play an important role in informing the future reform of the clinical negligence process in England.

**Systematic Review Registration:**

https://doi.org/10.17605/OSF.I0/6BP2N

## 1 Introduction

Improving patient experience is a prominent theme of the National Health Service (NHS) 10-Year Health Plan for England including the need to improve patient experience of clinical negligence claims (1). The National Audit Office, the United Kingdom's independent spending watchdog, estimated that only 4% of patients experiencing harm will make a claim for clinical negligence (2). This raises some questions about the factors that shape the choices of the remaining 96% who do not bring a claim for clinical negligence.

Over the last 10 years, there has been a significant change in the law and policy that supports providers of NHS healthcare in England to respond to patient harm. It has been reported that patients are significantly less likely to choose to bring a claim for clinical negligence if they receive an explanation of what happened, why it happened, how the problem will be corrected to prevent future harm and a sincere apology provided (3). Yet this assertion is based upon anecdotal knowledge rather than knowledge gained through the rigour of research. Exploring the factors that shape the choices of those who have suffered harm through rigorous inquiry would provide crucial insight into the perspectives and the post-incident response experienced by those affected by patient harm. Gaining such insight is important as it has the potential to inform policymakers and the future reform of the clinical negligence process in England.

This scoping review aimed to critically examine the limited body of empirical research and academic commentary exploring the factors that shape patient choices in bringing a claim for clinical negligence against the NHS in England and identify where gaps exist in current literature to inform future research directions. As the government in the United Kingdom has devolved its powers to the four nations, i.e., England, Wales, Scotland and Northern Ireland, each nation has its own policies, governance frameworks, legal frameworks and mechanisms for responding to patient harm, including the handling of claims for clinical negligence. Given this, and that England receives most clinical negligence claims in the United Kingdom, including the devolved nations, would introduce significant heterogeneity making comparisons and synthesis less meaningful. Therefore, this review is restricted to claims brought against the NHS in England.

## 2 Methods and analysis

Scoping review methodology aims to map and summarise the literature to date (4). To address the above aim, the Arksey and O'Malley six-stage framework for conducting scoping reviews (4) was adopted. To enhance the framework, the recommendations of Levac et al. (5) and the Joanna Briggs Institute (JBI) (6) were incorporated. The review was registered via the Open Science Framework (DOI.10.17605/OSF.IO/6BP2N) on 17 July 2024.

### 2.1 Stage 1: identifying the research question

The research question for this scoping review is: What are the factors identified by empirical research and academic commentary that shape patient choices in bringing a claim for clinical negligence against the NHS in England?

### 2.2 Stage 2: identifying relevant literature

#### 2.2.1 Search strategy

##### 2.2.1.1 Database search

Relevant published literature was identified by searching the peer-reviewed databases MEDLINE, Cumulative Index to Nursing and Allied Health Literature (CINAHL) and Westlaw. The search strategy included a combination of the following keywords, and where appropriate, corresponding MeSH terms, using Boolean operators such as AND and OR: National Health Service, choice, clinical negligence claim (see Table 1). The search was conducted on 18 July 2024 and re-ran on 22 March 2025 to identify any additional records.

**Table 1:** Complete MEDLINE and CINAHL search strategy.

No.	Keywords/search terms
#1	NHS OR ‘National Health Service’ OR healthcare
#2	Incident OR ‘serious incident’ OR ‘medical error’ OR ‘never event’ OR ‘avoidable injury’ OR ‘preventable injury’ OR ‘avoidable harm’ OR ‘preventable harm’ OR ‘patient safety incident’
#3	Motivation Or Decision OR Influence OR Deterrence OR Persuade OR Dissuade OR choice OR drivers
#4	‘Clinical negligence claim’ OR sue OR ‘litigation’ OR compensation OR claim
#5	#1 AND #2 AND #3 AND #4

### 2.3 Stage 3: study selection

Records retrieved from the search were included if they met the following criteria:

Considered factors that shape patient choices to bring a claim for clinical negligence against the NHS in England
Published in English

Records were excluded if they met any of the following criteria:

Considered factors that shape patient choices to bring a claim for clinical negligence against private healthcare providers
Considered factors that shape patient choices to bring a claim for clinical negligence against the NHS in Wales, Scotland or Northern Ireland.

No restriction was placed on publication date. The JBI guidance on scoping reviews (6) recommends that two or more reviewers screen articles independently. However, where this is not possible, for example, due to resource constraints, others have recommended that one reviewer conduct an independent review, with a second reviewer verifying a proportion of papers with a goal of 90% agreement (7). Guided by the above inclusion and exclusion criteria, the title and abstract were screened independently by one reviewer (NA). The full text of potentially relevant records was reviewed where uncertainty around eligibility existed. A second reviewer (GY) completed the same process by screening the title and abstract of 10% of the records retrieved and reviewed 10% of the records selected for full text review. A third reviewer (SG) was available to resolve any disagreements regarding the inclusion of texts. There was concordance of 100% between the two reviewers.

### 2.4 Stage 4: charting the data

A charting from was developed using parameters based on those described within the JBI Manual for Evidence Synthesis (6). This included classifying records as either research, academic commentary or policy. Development of the charting form was an iterative process as the review team continued to extract the data, became more familiar with the results extracted and updated the form accordingly. To ensure all relevant results were extracted, the charting form (Supplementary File S1) was piloted by different members of the review team and amended accordingly.

### 2.5 Stage 5: collating, summarising and reporting the results

To identify, analyse and report the themes within the data extracted from the included literature, the principles of thematic analysis outlined by Braun and Clarke (8) were drawn upon to identify initial codes and combined with the quantitative element of content analysis which counts the frequency of codes to identify recurring patterns (9). This required a five-phase process consisting of familiarisation with the literature (phase 1), generating initial codes relevant to the research question from each of the records reviewed (phase 2), identifying contextual themes (phase 3), measuring the frequency of initial codes (phase 4) and using the initial codes that occurred the most frequently and their contextual theme to iteratively and inductively derive the central themes of the data set (phase 5). This process was undertaken manually.

## 3 Results

### 3.1 Descriptive analysis

**Figure 1:**
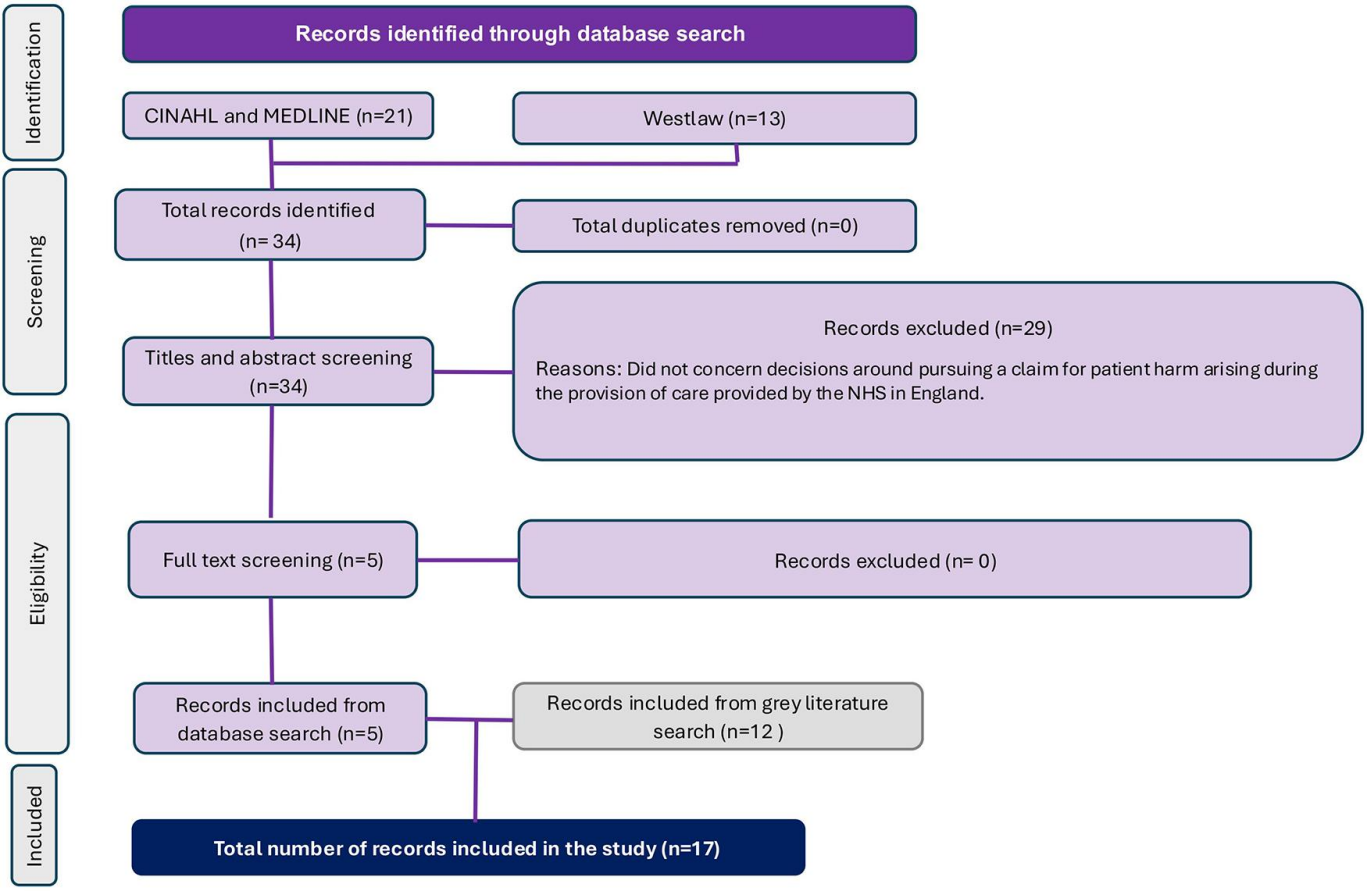
PRISMA flow chart.

The flow diagram (Figure 1) shows the results of the search, and the number of records found. A total of 34 records were identified, and 29 were excluded, leaving 5 records to be included in the scoping review. The bibliographic citations of the included literature were searched to identify further relevant records that fulfilled the inclusion criteria. This search yielded a further 12 records for inclusion. The total number of records included in the scoping review was 17.

**Figure 2:**
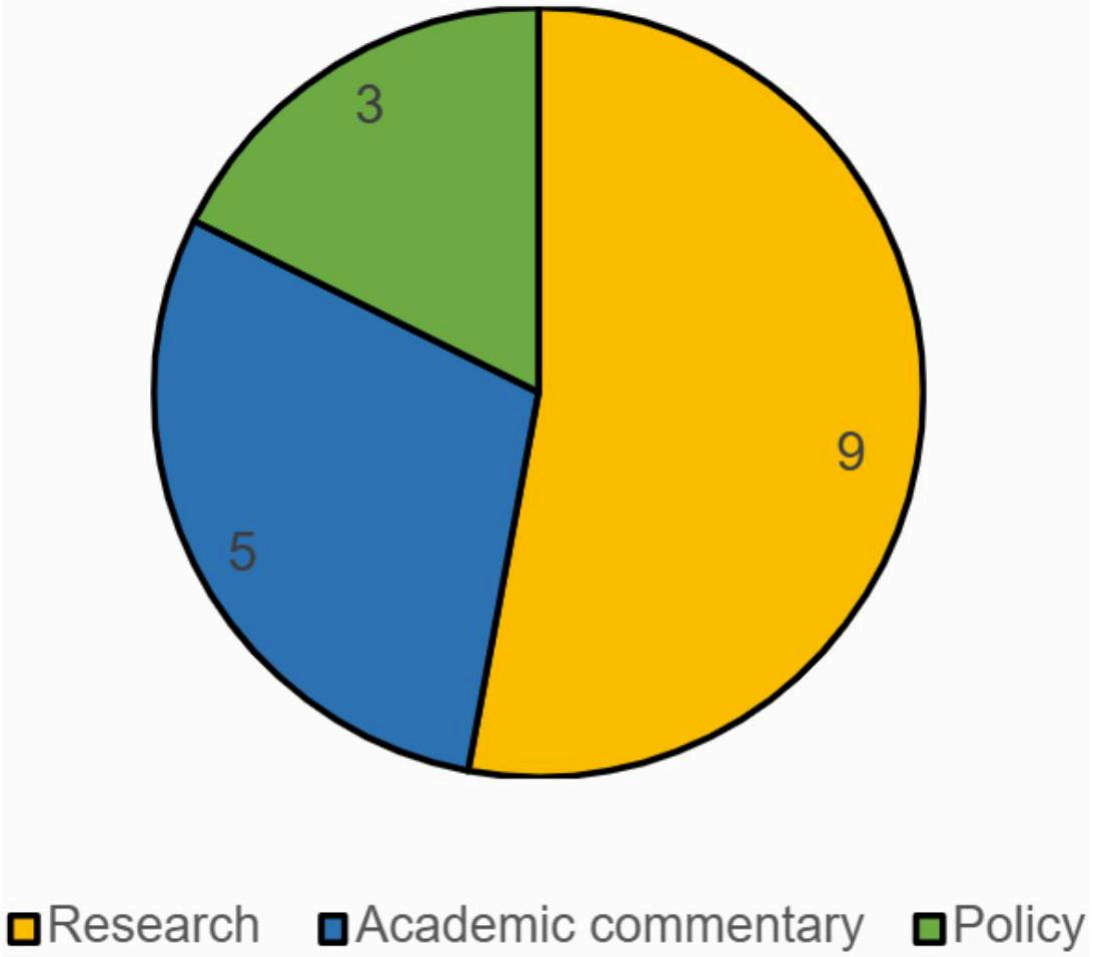
Volume of records by classification of publication.

**Figure 3:**
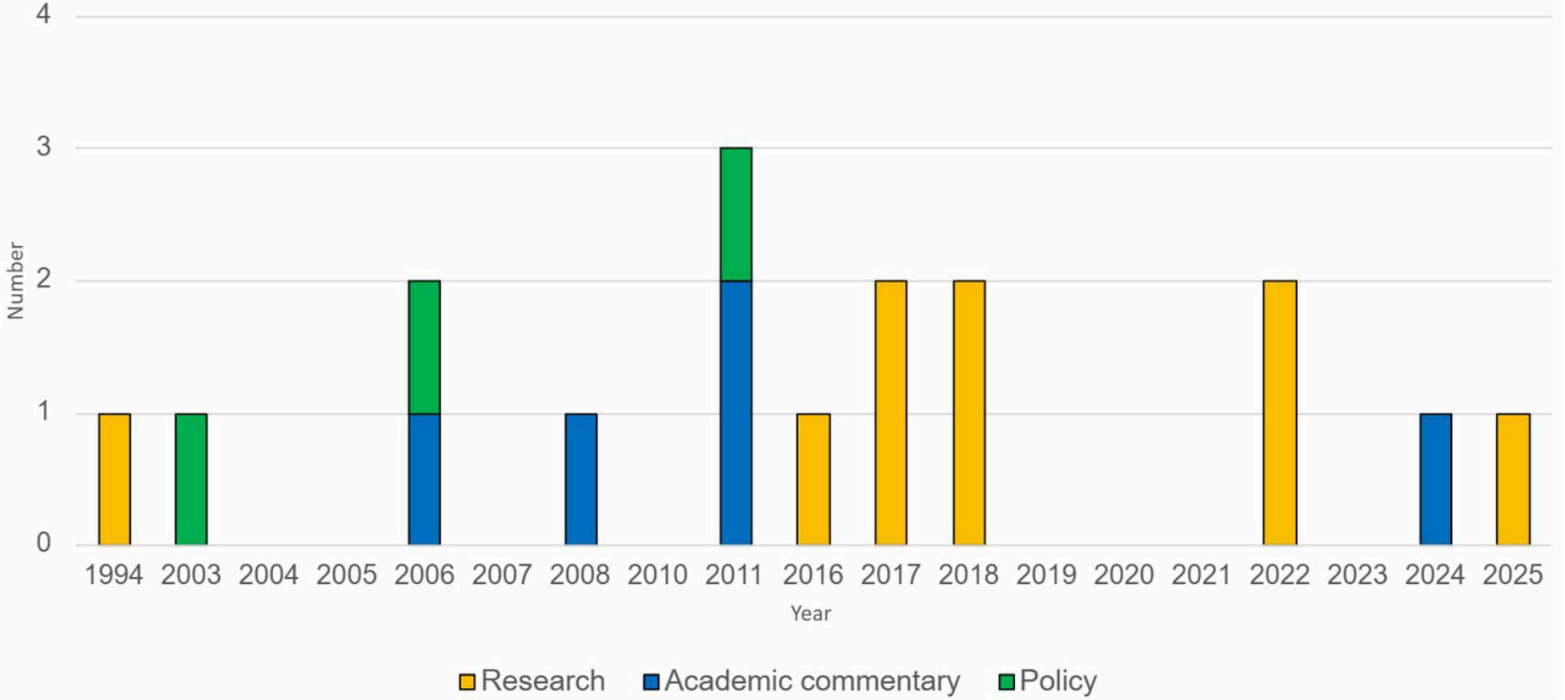
Distribution of publications by year and classification.

Charting of the records included in the scoping review involved capturing the year of publication, the classification of the record extracted, key findings including research characteristics, and following familiarisation, the initial codes relevant to each of the records (Supplementary File S1). Records were classified as either research, academic commentary or policy. Figure 2 shows the volume of records falling under each classification, and Figure 3 shows a graphical representation of the distribution of records by year of publication and classification.

### 3.2 Narrative synthesis

**Figure 4:**
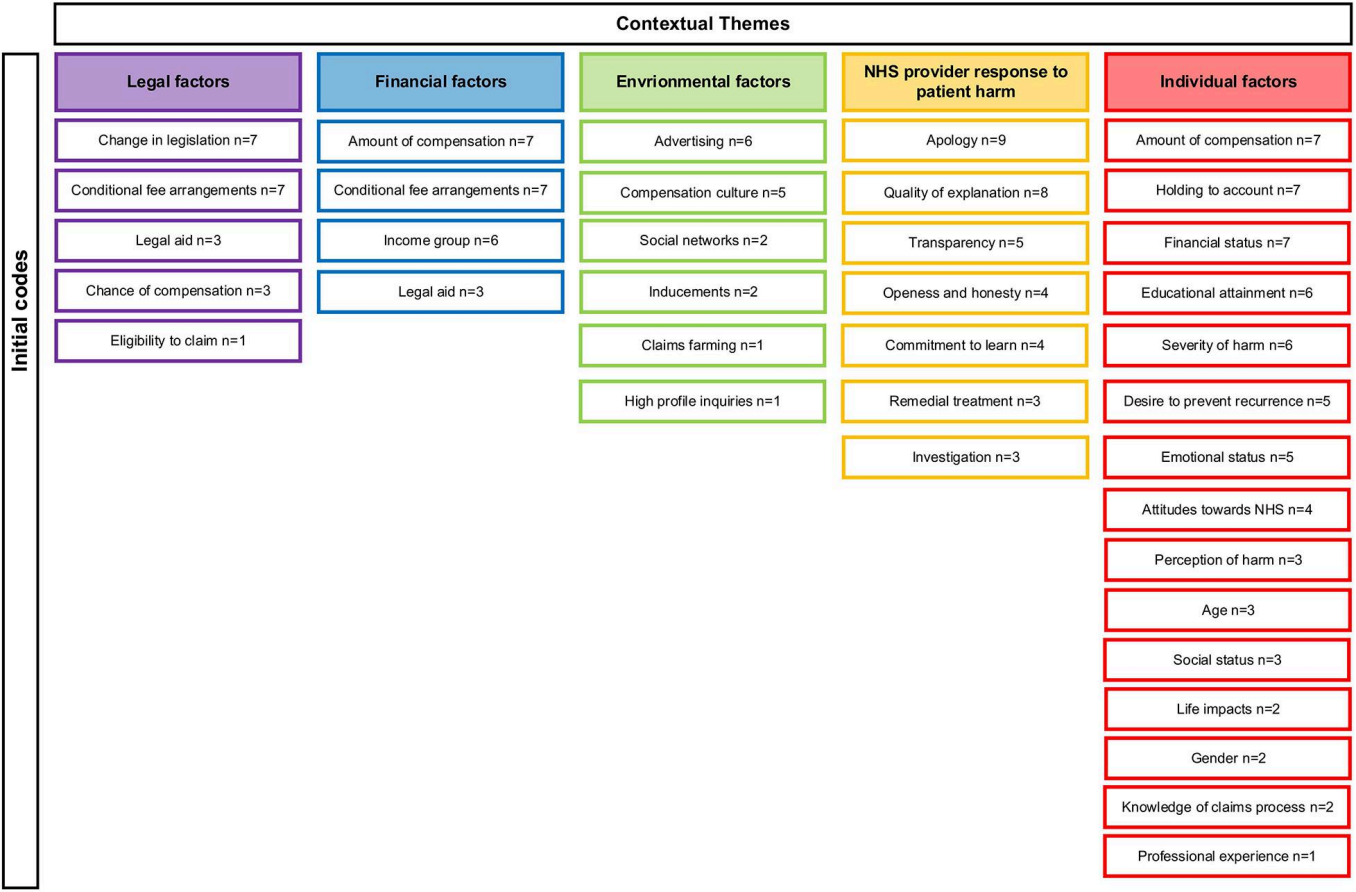
Summary of initial codes and contextual themes.

In total, 37 initial codes relevant to the research question were identified from the 17 records included in the scoping review. Through critical discussion with the research team, initial codes were categorised under five contextual themes: ‘legal factors’, ‘financial factors’, ‘environmental factors’, ‘NHS provider response to patient harm’ and ‘individual factors’. Figure 4 shows each of the initial codes and how these were categorised under each contextual theme. Of the five contextual themes generated, individual factors held the greatest number of initial codes (*n* = 15), followed by NHS provider response to patient harm (*n* = 7), and environmental factors (*n* = 6) (Figure 4). The records referenced under each initial code were tabulated and can be found in Supplementary File S1.

Four of the initial codes generated were categorised under more than one contextual theme. ‘Conditional fee arrangements’ and ‘legal aid’ were categorised under the contextual themes of legal factors and financial factors. ‘Income group’ and ‘amount of compensation’ were categorised under financial factors and individual factors. The narrative findings for each contextual theme, taking into account associated initial codes, are presented below.

### 3.3 Legal factors

This contextual theme describes legal frameworks associated with clinical negligence and how these shape choices to bring a claim for clinical negligence against the NHS in England. For example, records described how legal reforms resulted in changes to eligibility for legal aid and the introduction of conditional fee arrangements (CFAs) (2). The findings show these fundamental changes in the arrangements for access to justice impacted the type of claimant willing and able to bring claims against the NHS in England. For example, the reduced availability of legal aid led to a reduction in the proportion of lower social grades pursuing claims as conditional fee arrangements are not means tested (10). The willingness of a lawyer to take on a claim after reviewing its merits was described as a factor that directly impacts patient choice to bring a claim for clinical negligence against the NHS in England (10, 11).

### 3.4 Financial factors

This contextual theme describes the monetary matters that can shape patient choices to bring a claim for clinical negligence. The legal funding system within England was referenced across the records. The withdrawal of legal aid may have reduced the propensity for lower-income groups to make claims, but the growth of CFAs has made new options available to those in middle-income groups (10). Records described how conditional fee arrangements are generally lower value cases that incur lower costs compared with legal aid which is only available for complex birth injury cases (10, 12). However, to make the pursuit of a claim worthwhile for the lawyer and the claimant, a sufficiently large compensation reward needs to be available and was referenced as a factor that can shape choices to bring a claim for clinical negligence against the NHS in England. Claimants who have a lower value claim may be less likely to find a lawyer willing to take on their claim. Some claimants may choose not to progress with a claim if they deem the compensation reward to be low and not worthwhile pursuing (11, 13–15).

### 3.5 Environmental factors

Here, the cultural, political, and social factors that shape the context which subsequently affect the ultimate choice made by the individual are described within this contextual theme. The existence of a compensation culture was described within the records as a factor that influenced choices to bring a claim for clinical negligence as a societal perception exists that behind every accident, there is someone who is personally culpable and must pay (16, 17). High-profile patient safety concerns such as the Mid Staffordshire Inquiry were identified as being a factor that may shape the choice of patients to bring a claim for clinical negligence against the NHS in England. The role of advertising in shaping patient choice to bring a claim for clinical negligence claim against the NHS in England was also referenced as a factor across the records as this can increase awareness of the redress processes available (15, 16, 18).

### 3.6 NHS provider response to harm

This contextual theme describes the actions taken by NHS providers in the wake of patient harm. Being open and honest and providing an apology were key factors identified across the records as shaping whether those affected by patient harm chose to bring a claim for clinical negligence against the NHS in England (19, 20). The records indicated that where an NHS provider was open and honest, and an apology was provided, those affected by patient harm were less likely to bring a claim (14, 21). Conversely, where an NHS provider is not open and honest and does not provide an apology, those affected by patient harm are more likely to bring a claim for clinical negligence (22). The role of good communication, including providing good quality explanations, was described as a factor that can dissuade those affected by patient harm from bringing a claim for clinical negligence (12, 16, 19, 20).

### 3.7 Individual factors

This final contextual theme describes personal characteristics, knowledge, experience, attitudes and beliefs. Personal characteristics such as age, sex, level of qualification, household income and social status were all described as factors that can shape the choice of those affected by patient harm (10, 12). The severity of harm experienced by patients was described as a factor that can shape choices to pursue a claim for clinical negligence. Where disability has resulted, financial assistance may be required to make adaptations to the home and pay for care costs (10, 23, 24). The amount of compensation those affected by patient harm may potentially receive was also described as a factor that could shape choices to pursue a claim for clinical negligence (11). Records also described the value society places upon the NHS in England and the reluctance to bring a claim that will impact on financial resources (14, 15). However, wanting to hold the NHS to account was also described as a factor that could shape choices to bring a claim for clinical negligence against the NHS in England (11, 16, 17). The desire to prevent future harm and ensure learning was also described across the records as a factor that would shape the choices of those affected by patient harm to bring a claim for clinical negligence (13, 19, 20).

**Figure 5:**
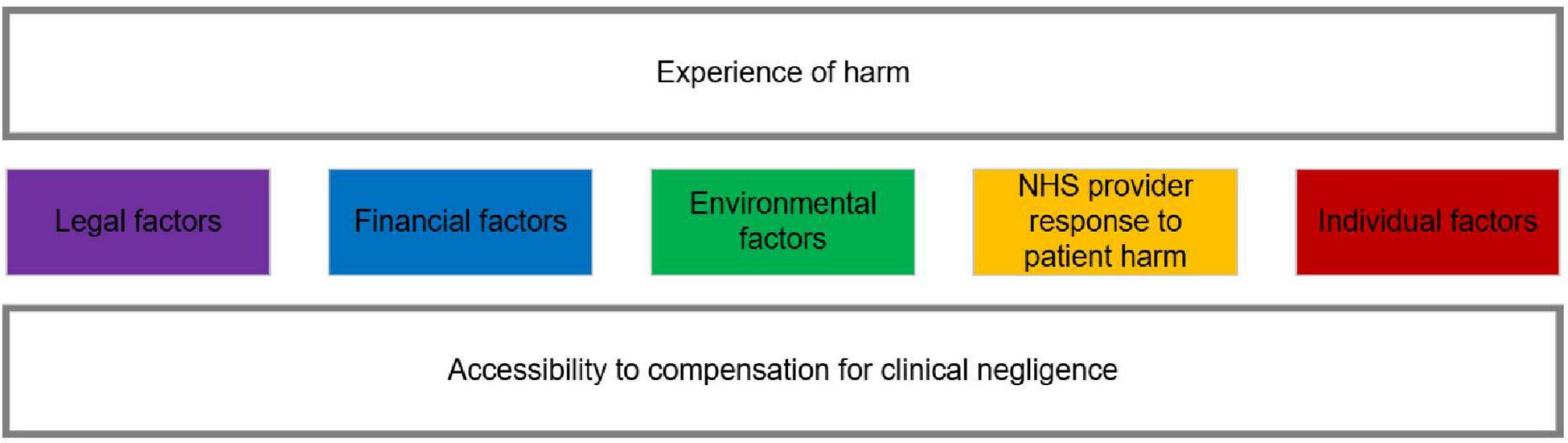
Main concepts related to contextual themes.

To complete the synthesis, the contextual themes were reviewed and refined to derive main concepts that encapsulated the essence of the extracted data through further critical discussion with the research team. Two main concepts were identified: ‘experience of harm’ and ‘accessibility to compensation for clinical negligence’ (Figure 5). Experience of harm describes factors such as the severity of harm, how NHS providers have responded to patient harm and how the beliefs of those affected by patient harm can shape choices to bring a claim for clinical negligence against the NHS in England. Accessibility to compensation for clinical negligence describes how the legal funding system in England, the merits of a claim and the knowledge of those affected by patient harm hold around the clinical negligence system can shape choices to bring a claim against the NHS in England. These conceptual themes are of relevance to each of the five contextual themes which are not mutually exclusive.

## 4 Discussion

This scoping review investigated the factors that shape the choices of patients to bring a claim for clinical negligence against the NHS in England. Two main concepts were identified: ‘experience of harm’ and ‘accessibility of compensation for clinical negligence’.

### 4.1 Experience of harm

Original research examining factors that shape patient choices to bring a claim for clinical negligence was conducted in the mid-1990s (19). To determine why patients and their relatives bring a claim for clinical negligence, 227 patients actively taking legal action against the NHS were surveyed. Analysis of the results identified that being severely affected by harm was the key factor for over 70% of respondents deciding to bring a claim for clinical negligence. They described themselves as having experienced physical problems, financial difficulties, breakdowns in family relationships, negative effects on social life and the inability to work. The results of the research also revealed the emotional impacts of harm with respondents describing feelings such as anger, bitterness, betrayal and humiliation (19).

To inform the Department of Health’s *Making Amends* report (20), a population survey was conducted in 2001 which identified that where the severity of harm was highest, 15% of those surveyed stated that they believed financial compensation to be the most appropriate remedy. In 2013, the population survey was repeated (24). Where the severity of harm was highest, the proportion of people stating they believed financial compensation was the most appropriate remedy increased to 18% in 2013 from 15% in 2001.

Severity of harm being a key factor that shapes the choices of patients to bring a claim for clinical negligence aligns with the findings of a discrete choice experiment methodology to determine UK public preference around different factors that influence the choice to make a claim for clinical negligence against the NHS (11). Using a series of hypothetical scenarios, the results of the study indicated that the probability of choosing to make a claim for clinical negligence was highest when the resulting harm was perceived as severe.

In addition to the severity of harm, research (19) identified a further four key factors that result in a choice to bring a claim for clinical negligence: holding to account those responsible for the harm, an explanation of why the harm occurred, preventing recurrence by improving standards of care and wanting an admission of negligence through compensation. Attitudes towards the NHS in England have changed dramatically in the last 5 years. The COVID-19 pandemic has impacted people's perceptions and expectations of the NHS. In 2024, just one in five British adults (21%) were ‘very’ or ‘quite’ satisfied with the way in which the NHS runs. This is the lowest level of satisfaction recorded since the survey began in 1983 and shows a steep decline of 39 percentage points since 2019 (25). How current societal attitudes towards the NHS in England are shaping the choices of those affected by patient harm to bring a claim for clinical negligence is currently unknown. This is an area requiring further research.

The 2001 population survey conducted to inform the *Making Amends* report (20) identified that where the severity of harm was high, 45% of those surveyed considered an apology and explanation to be the most appropriate response. However, this proportion decreased to 15% in the 2013 population survey (24). The reasons underpinning this shift in societal attitude were not explored by the authors. The research did identify that the proportion expressing a preference for support in dealing with the consequences of harm rose from 5% to 35%. Again, the authors did not explore what such support may consist of.

Research has identified that where the level of harm was considered low, the probability of choosing to make a claim for clinical negligence was less if an appropriate apology was provided, a detailed investigation was conducted and appropriate measures were implemented to prevent the harm from recurring (11). These findings align with the conclusions drawn from earlier research examining the motivations of those who had been successful in bringing a claim for clinical negligence (14). It can be inferred from these findings that a factor that may shape the choice of patients to bring a claim for clinical negligence could be the maturity of organisational safety culture, i.e., their values, attitudes, perceptions and behaviours regarding patient safety. This research was published prior to the Patient Safety Incident Framework (26) being implemented as part of the NHS standard contract (27). This new approach to responding to patient safety incidents aims to create a safer healthcare system by focusing on compassionate engagement with those affected, learning from incidents through system-based analysis rather than individual blame and implementing proportionate responses. How this significant shift in how NHS providers respond to patient harm will shape the choices of those affected by patient harm bring a claim for clinical negligence is unknown. This is an aspect requiring further inquiry through research.

### 4.2 Accessibility to compensation for clinical negligence

There is greater public awareness of the clinical negligence process in England (2). Analysis of the data extracted for this scoping review identified that the accessibility of this system shapes patient choice around bringing a claim for clinical negligence. Factors that affect accessibility include legal, financial, environmental and individual factors.

Before 2012, those seeking a claim for clinical negligence could access a funding stream that was means-tested and targeted those in a lower socio-economic classification. Access to legal aid funding was removed from most clinical negligence claims by the *Legal Aid and Sentencing and Punishment of Offenders Act 2012* (28). As a consequence of this reform, there is now minimal access to legal funding which has reduced the number of lower-income claimants bringing a claim for clinical negligence (10). To maintain access to justice and encourage solicitors to continue taking on claims for clinical negligence, the use of outcome-based fees such as CFAs was permitted and was not means-tested. This formalised ‘no win no fee’ arrangements for solicitors and enabled them to target high-volume, middle-income groups and assess their claims in terms of case strength and value. Furthermore, the creation of ‘After the Event’ (ATE) insurance meant that defendant costs would be covered in the event of a claim not being successful. These legal reforms resulted in greater access to justice for those in middle-income groups.

Research conducted in 2016 explored changes associated with claims that had occurred because of the shift in funding opportunities for clinical negligence claims in England and Wales in the preceding 15 years (10). This included evaluating changes in the demographic of claimants. Drawing on data sets including freedom of information requests and survey results, the researchers explored how legal aid, CFAs and legal insurance expenses affected the type of claimant willing and able to bring a claim against the NHS in the preceding 15 years. Although the proportion of people experiencing adverse events and subsequently bringing a claim for clinical negligence changed very little between 2001 and 2013, there did appear to be a reduction in the propensity for lower-income groups to bring a claim for clinical negligence following the withdrawal of legal aid. The reasons for this observation were not explored by the researchers in any depth but may suggest that household income, financial capability and the ability to pay legal fees should a claim not result in compensation are factors that may shape choices around bringing a claim for clinical negligence. These findings also indicate how legal reform and financial capability potentially create barriers to accessing compensation and are factors that may shape choices around bringing a claim for clinical negligence. Understanding the relationship between social determinants of health, health inequalities and the factors that shape the choices of those affected by patient harm, are areas requiring further inquiry through research.

Analysis of records extracted for this scoping review identified environmental factors that can shape patient choice in bringing a claim for clinical negligence. The advertising strategies of clinical negligence lawyers were cited across records extracted for this scoping review (14–16, 18, 29). Whilst law firms must conform to codes of advertising, the review highlighted the opinion that these were not robust enough permitting advertising that suggests that people can easily claim compensation for the most minor of incidents contributing to a compensation culture being established (17). Whilst advertising may prompt a claim for clinical negligence to be considered, the anticipated length of time to manage the claim was a factor that could dissuade those who have begun to consider bringing a claim for clinical negligence from continuing (11). None of the records extracted for this scoping review considered the role of social media or Artificial Intelligence (AI) in shaping patient choices in bringing a claim for clinical negligence. This is an area for further research.

Analysis of the records extracted for the scoping review identified individual factors that may shape patient choices in bringing a claim for clinical negligence. The perception that there are generational differences in attitudes towards bringing a claim for clinical negligence with older people being reluctant to bring a claim has been identified as a factor (2). In contrast, age has been found to not alter the probability associated with choosing to bring a claim for clinical negligence (16). This was also found to be the case for gender (24). This finding aligns with other research which found gender was not a variable that significantly influenced the likelihood of a patient choosing to bring a claim (10). Whether other socio-demographic variables such as race, ethnicity, religious affiliation, education, employment status and disability shape patient choice in bringing a claim for clinical negligence was not considered in any of the data extracted for this scoping review. To gain greater insight into all factors at play, it is important to explore how broader socio-demographic variables shape patient choices in bringing a claim for clinical negligence.

Whether the educational attainment of those experiencing harm shapes patient choice to bring a claim for clinical negligence was to some extent considered within the records extracted for the scoping review (11, 14, 18–20). *Making Amends* (20) identified an imbalance between the knowledge of those bringing a claim for clinical negligence and those defending the claim. It was considered that this imbalance placed those choosing to bring a claim for clinical negligence at a disadvantage. None of the records extracted for the scoping review considered whether the educational attainment of those experiencing harm shapes patient choices in bringing a claim for clinical negligence although there was an assumption that the knowledge and expertise of patients is less than that of clinicians albeit valuable (19).

## 5 Strengths and limitations

There are strengths and limitations associated with this scoping review. Utilising the scoping review methodology has enabled the systematic search of the MEDLINE, CINAHL and Westlaw databases to systematically analyse existing records and identify relevant grey literature to answer the research question. Synthesis of the records has enabled the identification of gaps in current knowledge and understanding and provided direction for future research.

There is no formal assessment of the methodological quality of the literature included in a scoping review (30), and therefore, studies of low quality may be included. However, a benefit of the scoping review methodology is that the review question is broader in nature and outlines all literature regardless of quality, which allows a wider ranging and more contextual overview (31).

## 6 Conclusion

Only 4% of people experiencing harm will make a claim for clinical negligence (2). This review investigated the factors identified by research and academic commentary as shaping patient choices in bringing a claim for clinical negligence against the NHS in England. It found that response to patient harm can shape patient choice in bringing a claim for clinical negligence. Where a high-quality response to patient harm is provided, it is possible that those affected may be dissuaded from bringing a claim for clinical negligence. The findings of this scoping review suggest that where harm severity is high, it is the need for financial support to put those affected back in the position that they would have been had the harm not occurred can shape patient choice to bring a claim for clinical negligence rather than the response of those responsible for the harm. The records identified for this scoping review gave limited consideration to the current factors shaping the choices of the remaining 96% to not bring a claim for clinical negligence.

Over the last 10 years, there have been significant changes to the law and policy that guide how providers of NHS healthcare in England respond to patient harm. Greater attention is also being given to social determinants of health and inequalities in healthcare. This scoping review has identified the limited consideration given to how current law and policy, organisational cultures, social determinants of health and health inequalities are currently shaping patient choices to bring a claim for clinical negligence. The need to examine the role of social media or AI in shaping patient choices in bringing a claim for clinical negligence has also been identified. Future research should explore these factors by employing qualitative methodologies such as semi-structured interviews and surveys. This is vital to improve patient experience of the clinical negligence process in England and has the potential to play an important role in informing the future reform of the clinical negligence process in England.

## Data Availability

The original contributions presented in the study are included in the article/Supplementary Material; further inquiries can be directed to the corresponding author.
