# Supplementary material for: Understanding the factors that shape patient choices in bringing a claim for clinical negligence against the NHS in England: a scoping review
Source: Front Health Serv. 2026 Jan 8;5:1696964. doi: 10.3389/frhs.2025.1696964 (PMC12823948; doi:10.3389/frhs.2025.1696964)
Supplement: Supplementary file 2 [file Table2.pdf]

Table 1: Records relating to factors that influence the decisions of those affected by patient harm to pursue a claim for clinical negligence grouped into initial codes and the contextual theme of legal factors.

Initial Code	Frequency	Records
Change in legislation	N=7	Gray, A., Fenn, P., Rickman, N. and Vencappa, D. (2016); National Audit Office (2017); Birks, Y. (2018); Parliament. House of Commons (2006); Oliphant, K., Lewis, R. and Morris, A. (2006); Morris, A. (2011); House of Commons Health Committee (2011);
Conditional fee arrangements	N=7	Gray, A., Fenn, P., Rickman, N. and Vencappa, D. (2016); National Audit Office (2017); Birks, Y. (2018); Parliament. House of Commons (2006); Oliphant, K., Lewis, R. and Morris, A. (2006); Morris, A. (2011); House of Commons Health Committee (2011);
Legal aid	N=3	Gray, A., Fenn, P., Rickman, N. and Vencappa, D. (2016); Parliament. House of Commons (2006); Oliphant, K., Lewis, R. and Morris, A. (2006);
Chance of compensation	N=3	Rowen, D. et. al. (2022); Birks, Y. (2018); Wright, J. and Opperman, G. (2008);
Eligibility to claim compensation	N=1	Rowen, D. et. al. (2022);

Table 2: Records relating to factors that influence the decisions of those affected by patient harm to pursue a claim for clinical negligence grouped into initial codes and the contextual theme of financial factors.

Initial Code	Frequency	Records
Amount of compensation	N=7	Rowen, D. et. al. (2022); Cave, E. (2011); Gray, A., Fenn, P., Rickman, N. and Vencappa, D. (2017); NHS Resolution (2018); Vincent, C., Phillips, A. and Young, M. (1994); Birks, Y. (2018); Parliament. House of Commons (2006);
Conditional fee arrangements	N=7	Gray, A., Fenn, P., Rickman, N. and Vencappa, D. (2016); National Audit Office (2017); Birks, Y. (2018); Parliament. House of Commons (2006); Oliphant, K., Lewis, R. and Morris, A. (2006); Morris, A. (2011); House of Commons Health Committee (2011);
Income group	N=6	Cave, E. (2011); Gray, A., Fenn, P., Rickman, N. and Vencappa, D. (2017); NHS Resolution (2018); Gray, A., Fenn, P., Rickman, N. and Vencappa, D. (2016); Birks, Y. (2018); Parliament. House of Commons (2006);
Legal aid	N=3	Gray, A., Fenn, P., Rickman, N. and Vencappa, D. (2016); Parliament. House of Commons (2006); Oliphant, K., Lewis, R. and Morris, A. (2006);

Table 3: Records relating to factors that influence the decisions of those affected by patient harm to pursue a claim for clinical negligence grouped into initial codes and the contextual theme of environmental factors.

Initial Code	Frequency	Records
Advertising	N=6	Carter, A. et. al. (2022); NHS Resolution (2018); Parliament. House of Commons (2006); Oliphant, K., Lewis, R. and Morris, A. (2006); Morris, A. (2011); House of Commons Health Committee (2011);
Compensation culture	N=5	Department of Health (2003); Birks, Y. (2018); Parliament. House of Commons (2006); Oliphant, K., Lewis, R. and Morris, A. (2006); Morris, A. (2011);
Social networks	N=2	NHS Resolution (2018); Birks, Y. (2018);
Inducements	N=2	Morris, A. (2011); House of Commons Health Committee (2011);
Claims farming	N=1	Parliament. House of Commons (2006);
High profile inquiries	N=1	National Audit Office (2017);

Table 4: Records relating to factors that influence the decisions of those affected by patient harm to pursue a claim for clinical negligence grouped into initial codes and the contextual theme of NHS provider response to patient harm.

Initial Code	Frequency	Records
Apology	N=9	Rowen, D. et. al. (2022); Cave, E. (2011); NHS Resolution (2018); Vincent, C., Phillips, A. and Young, M. (1994); Department of Health (2003); Birks, Y. (2018); Parliament. House of Commons (2006); Wright, J. and Opperman, G. (2008); Ramsay, L. et. al. (2025);
Quality of explanation	N=8	Cave, E. (2011); NHS Resolution (2018); National Audit Office (2017); Vincent, C., Phillips, A. and Young, M. (1994); Department of Health (2003); Birks, Y. (2018); Parliament. House of Commons (2006); Ramsay, L. et. al. (2025);
Transparency	N=5	Rowen, D. et. al. (2022); National Audit Office (2017); Vincent, C., Phillips, A. and Young, M. (1994); Birks, Y. (2018); Ramsay, L. et. al. (2025);
Openness and honesty	N=4	NHS Resolution (2018); Birks, Y. (2018); Parliament. House of Commons (2006); O'Dwyer, L. (2024);
Commitment to learn	N=4	Cave, E.(2011); NHS Resolution (2018); Birks, Y. (2018); O'Dwyer, L. (2024);
Remedial treatment	N=3	Vincent, C., Phillips, A. and Young, M. (1994); Department of Health (2003); Parliament. House of Commons (2006);
Investigation	N=3	Rowen, D. et. al. (2022); NHS Resolution (2018); Ramsay, L. et. al. (2025);

Table 5: Records relating to factors that influence the decisions of those affected by patient harm to pursue a claim for clinical negligence grouped into initial codes and the contextual theme of individual factors.

Initial Code	Frequency	Records
Amount of compensation	N=7	Rowen, D. et. al. (2022); Cave, E. (2011); Gray, A., Fenn, P., Rickman, N. and Vencappa, D. (2017); NHS Resolution (2018); Vincent, C., Phillips, A. and Young, M. (1994); Birks, Y. (2018); Parliament. House of Commons (2006);
Holding to account	N=7	Rowen, D. et. al. (2022); Cave, E. (2011); NHS Resolution (2018); Vincent, C., Phillips, A. and Young, M. (1994); Department of Health (2003); Birks, Y. (2018); O'Dwyer, L. (2024);
Financial status	N=7	Cave, E. (2011); Gray, A., Fenn, P., Rickman, N. and Vencappa, D.(2017); NHS Resolution (2018); Gray, A., Fenn, P., Rickman, N. and Vencappa, D. (2016);Birks, Y. (2018); Parliament. House of Commons (2006); Ramsay, L. et. al. (2025);
Educational attainment	N=6	Rowen, D. et. al. (2022); NHS Resolution (2018); Vincent, C., Phillips, A. and Young, M. (1994); Department of Health (2003); Parliament. House of Commons (2006); Ramsay, L. et. al. (2025);
Severity of harm	N=6	Rowen, D. et. al. (2022); Gray, A., Fenn, P., Rickman, N. and Vencappa, D.(2017); Vincent, C., Phillips, A. and Young, M. (1994); Department of Health (2003); Birks, Y. (2018); Ramsay, L. et. al. (2025);
Desire to prevent recurrence	N=5	Cave, E. (2011); NHS Resolution (2018); Vincent, C., Phillips, A. and Young, M. (1994); Department of Health (2003); Birks, Y. (2018);
Emotional status	N=5	Rowen, D. et. al. (2022); NHS Resolution (2018); Vincent, C., Phillips, A. and Young, M. (1994); Birks, Y. (2018); Ramsay, L. et. al. (2025);

Table 5: Records relating to factors that influence the decisions of those affected by patient harm to pursue a claim for clinical negligence grouped into initial codes and the contextual theme of individual factors.

Initial Code	Frequency	Records
Attitudes towards NHS	N=4	Rowen, D. et. al. (2022); National Audit Office (2017); Birks, Y. (2018); Oliphant, K., Lewis, R. and Morris, A. (2006);
Perception of harm	N=3	Vincent, C., Phillips, A. and Young, M. (1994); Department of Health (2003); Birks, Y. (2018);
Age	N=3	Gray, A., Fenn, P., Rickman, N. and Vencappa, D. (2017); Gray, A., Fenn, P., Rickman, N. and Vencappa, D. (2016); Birks, Y. (2018);
Social status	N=3	Gray, A., Fenn, P., Rickman, N. and Vencappa, D. (2017); Gray, A., Fenn, P., Rickman, N. and Vencappa, D. (2016); Ramsay, L. et. al. (2025);
Life impacts	N=2	Vincent, C., Phillips, A. and Young, M. (1994); Ramsay, L. et. al. (2025);
Gender	N=2	Gray, A., Fenn, P., Rickman, N. and Vencappa, D. (2017); Gray, A., Fenn, P., Rickman, N. and Vencappa, D. (2016);
Knowledge of claims process	N=2	National Audit Office (2017); House of Commons Health Committe (2011);
Professional experience	N=1	NHS Resolution (2018);
